# Autograft microskin combined with adipose-derived stem cell enhances wound healing in a full-thickness skin defect mouse model

**DOI:** 10.1186/s13287-019-1389-4

**Published:** 2019-08-30

**Authors:** Yuansen Luo, Xiaoyou Yi, Tangzhao Liang, Shihai Jiang, Ronghan He, Ying Hu, Li Bai, Chunmei Wang, Kun Wang, Lei Zhu

**Affiliations:** 1 Department of Plastic and Aesthetic Surgery, The Third Affiliated Hospital of Sun Yat-sen University, No.600 Tianhe Road, Tianhe District, Guangzhou, 510630 China; 2 Department of Orthopedics Surgery, Tungwah Hospital of Sun Yat-sen University, 523110 Dongguan, China; 3 Department of Joint and Trauma Surgery, the Third Affiliated Hospital of Sun Yat-sen University, 510630 Guangzhou, China; 4 Department of Plastic and Aesthetic Surgery, Dermatology Hospital of Southern Medical University, 510630 Guangzhou, China

**Keywords:** Adipose-derived stem cell, Microskin, Secretome, Full-thickness skin defect, Wound healing

## Abstract

**Objective:**

Autograft microskin transplantation has been widely used as a skin graft therapy in full-thickness skin defect. However, skin grafting failure can lead to a pathological delay wound healing due to a poor vascularization bed. Considering the active role of adipose-derived stem cell (ADSC) in promoting angiogenesis, we intend to investigate the efficacy of autograft microskin combined with ADSC transplantation for facilitating wound healing in a full-thickness skin defect mouse model.

**Material and methods:**

An in vivo full-thickness skin defect mouse model was used to evaluate the contribution of transplantation microskin and ADSC in wound healing. The angiogenesis was detected by immunohistochemistry staining. In vitro paracrine signaling pathway was evaluated by protein array and Gene Ontology, Kyoto Encyclopedia of Genes and Genomes pathway, and protein-protein interaction network analysis.

**Results:**

Co-transplantation of microskin and ADSC potentiated the wound healing with better epithelization, smaller scar thickness, and higher angiogenesis (CD31) in the subcutaneous layer. We found both EGF and VEGF cytokines were secreted by microskin in vitro. Additionally, secretome proteomic analysis in a co-culture system of microskin and ADSC revealed that ADSC could secrete a wide range of important molecules to form a reacting network with microskin, including VEGF, IL-6, EGF, uPAR, MCP-3, G-CSF, and Tie-2, which most likely supported the angiogenesis effect as observed.

**Conclusion:**

Overall, we concluded that the use of ADSC partially modulates microskin function and enhances wound healing by promoting angiogenesis in a full-thickness skin defect mouse model.

**Electronic supplementary material:**

The online version of this article (10.1186/s13287-019-1389-4) contains supplementary material, which is available to authorized users.

## Background

Wound healing is a remarkably complex, and continuous process consisted of hemostasis and coagulation, inflammation, proliferation, and wound repairing with scar tissue formation [1]. Inappropriate management of wound care would result in a negative contribution to the healing process and potential complications, such as delay or non-healing wounds. Microskin grafting is a method of laying small sheets of the skin graft on the cutaneous wound to enhance wound healing which has been widely used as skin graft therapy in developing countries [2]. The procedure is simple and economical which has been approved successfully in full-thickness skin defect. However, there is a limitation of microskin grafting such as lack of neovascularization, keloid scar formation, and failure of transplantation due to poor wound bed and ischemia-reperfusion (IR) [3]. Therefore, burn surgeons face a considerable challenge as to how to enhance the effectiveness of microskin grafting.

In recent years, adipose-derived stem cell (ADSC) application, as a stem cell-based therapy, has been proven to promote tissue regeneration in chronic and non-healing wounds owing to their differentiation and paracrine effects [4]. ADSCs have attracted focus widely because they can be harvested with minimal invasiveness. Early in vitro studies reported that ADSCs could secrete various bioactive factors such as vascular endothelial growth factor (VEGF), epidermal growth factor (EGF), and hepatocyte growth factor (HGF), which were beneficial to enhance endothelial cell (EC) function and promote angiogenesis [5]. Moreover, extracellular vesicles (EVs) released from ADSCs can stimulate proliferation of human microvascular endothelial cells (HMECs) and enhance pro-angiogenic function [6]. These data show the ability of ADSCs to enhance neovascularization, and paracrine function may play a critical role in angiogenesis. In the model of extended inferior epigastric artery skin flap in rats, treatment with ADSCs increased flap survival by enhancing angiogenic response and improving blood perfusion [7]. Besides, significantly accelerate neovascularization has been found in venous congested skin graft of rabbit model treated with ADSCs [8]. Owing to its paracrine function, ADSCs have been widely applied as a new therapy to skin wound healing and skin graft in recent years.

Although microskin grafting is the main method of massive skin defects, there are still several problems we need to solve as previously discussed, such as insufficient angiogenesis. We hypothesized that the paracrine function of ADSCs might enhance angiogenesis promotion in microskin grafting. Thus, we aim to explore if microskin in a combination of ADSCs could promote the wound healing of full-thickness skin defects and conquer the limitation of microskin grafting. In our study, a number of cytokines secreted by the co-culture system of microskin and ADSCs, including vascular endothelial growth factor (VEGF), interleukin 6 (IL-6), epidermal growth factor (EGF), urokinase plasminogen activator receptor (uPAR), monocyte chemotactic protein-3 (MCP-3), granulocyte colony-stimulating factor (G-CSF), and tyrosine kinase with immunoglobulin-like and EGF-like domains 2 (Tie-2), were identified by high-throughput protein array. These cytokines contribute to angiogenesis and promote wound healing. Therefore, our in vivo and in vitro study suggests that the combination of microskin and ADSCs could be a promising therapy to promote wound healing of full-thickness skin defect.

## Materials and methods

### Animals

The animal protocol was approved by the Institutional Animal Research Committee Approval of Sun Yat-sen University. All the animals were purchased from the Animal Center for Medical Experiment of Guangdong. This study has been conducted under the guideline of the Guide for the Care and Use of Laboratory Animals.

### Isolation, culture, and characterization of adipose-derived stem cell

ADSC extraction was performed as described by Zuk et al. [9]. The inguinal subcutaneous fat was isolated from the Balb/c mice (male, 12 weeks old). The adipose was washed with phosphate-buffered saline (PBS, Gibco, USA) consists of 1% penicillin-streptomycin Solution (Keygen, Jiangsu, China) three times, minced with scissors into pieces less than 1-mm diameter, washed with PBS, and centrifuge for 5 min at 400*g* three times. For the floating tissue, a threefold volume of 1% type I collagenase (Gibco, USA) was added, and the admixture was then digested in 37 °C water bath and shaken gently every 5 min for 45 min. The deposit was suspended with culture medium and pass through a 70-μm filter to removed undigested tissue. The pellets were resuspended with culture medium to a final concentration of 5 × 10^6^cells/ml; then, the cells were placed in a 37 °C incubator supplied with 5% CO_2_ and 95% humidity. The medium was changed every 2 days. The third or fourth passage cells were used for various experiments.

After the third or fourth passage, cells were harvested and applied to characterize the CD markers of mesenchymal stem cells. The protocols were adopted and followed by other previously published studies [10]. Briefly, 50 μl of cell suspension was incubated with a fluorochrome-conjugated monoclonal antibody for 1 h in the dark at room temperature, washed three times with PBS, and analyzed using a FACS Calibur flow cytometer (Becton Dickinson, San Jose, CA). The antibodies used in the experiments were HLA-DR, CD11b, CD19, CD34, CD45, CD73, CD90, CD105 (Abcam, USA). Mouse fluorochrome-conjugated isotype control IgG antibodies (Abcam, USA) were used in the experiments as a negative labeling control.

To analyze cell differentiation abilities of adipogenic, osteogenesis, and chondrogenic, ADSCs were cultured in adipogenic differentiation medium for 2 weeks, osteogenesis differentiation medium for 3 weeks, and chondrogenic differentiation medium for 4 weeks (ScienceCell, USA). Cells were fixed with 4% paraformaldehyde in PBS for 1 h at room temperature and stained with Oil Red O, Alizarin Red, and Alcian blue (Sigma) solution. The results were observed under a phase contrast microscope (Nikon, Japan).

### Fabrication of microskin

Preparation of microskin was performed as described by Zhang et al. [11], with a bit of modification. Balb/c mice (male, 12 weeks old) weighing approximately 22 g were used in this experiment. Their backs were shaved and wiped with 75% ethyl alcohol after intraperitoneal injection of 50 mg/kg sodium pentobarbital for anesthesia. Then, a piece of full-thickness skin (2 cm × 3 cm) was removed from the subjected mouse and cut into 5 mm × 5 mm with a scissor. The debris of the skin was then immersed in PBS and washed three times. For the debris of the skin, a threefold volume of 0.25% dispase was added and incubated at 4 °C overnight to detach the dermis and the epidermis. The epidermis was cut into less than 1-mm diameter (microskin) and washed with PBS.

### In vivo mouse wound healing model

Thirty Balb/c mice (male, 8 weeks old), weighing 22 ± 4 g, were used in this experiment. Mice were housed in the environment without specific pathogen and free to access standard food and water with 12-h photoperiods. Before the surgical procedure, all the mice accepted anesthesia by 50 mg/kg sodium pentobarbital by intraperitoneal injection. Their dorsal surface, including the surgical area, was shaved exhaustively and wiped with 75% ethyl alcohol twice. Mice were randomized into two parts (*n* = 15 each) depending on the processing treatment after surgery. Two round shape of the full-thickness wound (1.2-cm diameter) were created in the middle of the back of each mouse. The wounds were photographed by digital camera immediately. According to local treatment used for each mouse, one wound was transplanted 1/4 area autologous microskin evenly (as previously described) and 30 μl PBS (MS group), the other was transplanted 30 μl PBS as a negative control (control group). A coupled mouse was manipulated similarly, and one wound was transplanted 1/4 area autologous microskin and 30 μl ADSC with 1 × 10^5^ cells (MS+ADSC group), the other was transplant 30 μl ADSC with 1 × 10^5^ cells as control (ADSC group). The ADSCs were prepared before the surgery and injected into the surface of the wound area. A polyethylene collar was stitched to the adjacent skin to retain the margin of the wound. The mice were administered with sodium salicylate (150 mg/kg) for pain control and antibiotic for the following 2 days. At the time point of 7 days and 14 days after surgery, the wounded skin was photographed. To evaluate the therapeutic effect of each treatment, we performed a photograph of the wounded skin by a digital camera at 0 days, 7 days, and 14 days post-treatment and analyze the data by ImageJ (NIH, Bethesda, MD) software. All the measurements of the wound area and wound contraction were followed as previous studies [12]. For the wound area studies, we defined that the actual wound area was open wound area and it was calculated using the following formula:

$$ \%\mathrm{wound}\ \mathrm{area}=\left({\mathrm{W}}_0-{\mathrm{W}}_1-{\mathrm{W}}_2\right)/{\mathrm{W}}_0\times 100\% $$

For the wound contraction studies, it was calculated using the following formula:

$$ \%\mathrm{wound}\ \mathrm{contraction}=\left({\mathrm{W}}_0-{\mathrm{W}}_1\right)/{\mathrm{W}}_0\times 100\% $$

where W_0_ was the original wound area on day 0, W_1_ was the open wound area at day 7 and day 14, and W_2_ was the area of microskin adhering to the skin or re-epithelialization area. The sum of contraction area, re-epithelialization area or microskin area, and open wound areas equal 100% of the original wound size. Meanwhile, half of the mice were sacrificed by CO_2_ asphyxiation and carefully harvested the wounded skin for histological analysis at each time point.

### Histochemistry and immunohistochemistry

For histochemistry and immunohistochemistry analysis, the wounded skins taken at each time point were excised and fixed in 4% paraformaldehyde, embedded in paraffin, and sectioned vertically into 4-μm-thick sections. For histological observations, representative sections were stained for hematoxylin and eosin (H&E) following conventional protocols. Alpha smooth muscle actin (a-SMA), CD31, and vascular endothelial growth factor (VEGF) were chosen to perform immunohistochemistry to evaluate fibrosis and neovascularization following routine protocols [13]. Briefly, the tissue sections were performed in citrate-based antigen retrieval for 15 min and blocked with normal goat serum for 30 min. Then, the sections were incubated with anti-CD31 (1:100; Abcam, UK), anti-VEGF (1:100; Abcam, UK), and anti-alpha smooth muscle actin (anti-α-SMA, 1:150; Abcam, UK) antibodies at 4 °C overnight, separately. After washing with PBS, the sections were developed with DAB and counterstained with hematoxylin. The sections were analyzed and images acquired with an upright optical microscope (Nikon, Japan). Neovascularization at the wound sites was detected by CD31 staining. Microphotographs were captured, and quantification of CD31-positive (+) blood vessels was performed in ten random fields per section. Only the blood vessels, which have a diameter of 2–10 μm, were counted as one vessel [14]. For the measurement of scar thickness (the distance from the epidermal-dermal junction down to the panniculus carnosus, Additional file 4: Figure S3A), five random distances with equal gap (1000 μm) of scar thickness were measured in the wound area which are determined on three H&E staining sections each group using ImageJ software [15].

### Interaction detection by co-culture of microskin and ADSC, ADSC, and fibroblasts

It is reported that ADSCs could differentiate into keratinocyte and endotheliocyte cells in vitro [16, 17]. To investigate whether ADSCs differentiated into keratinocyte or endotheliocyte cells while co-culturing with microskin, we performed a co-culture system, which was adopted and followed by previously published studies [18]. An 8-μm micropore, 6-well Transwell plate (Millipore, USA) was used to co-culture microskin and ADSC. 1 × 10^5^cells/ml ADSC was seeded in the lower chamber, and microskin about 1-cm^2^ epidermis area was put in the upper chamber. The Transwell system was supplied by DMEM medium with 2% FBS. After incubated for 7 days and 14 days, total RNA and protein of ADSC were extracted and preserved in − 80 °C for further research. Cultured ADSC supplied by DMEM medium with 2% FBS was taken as the control group. qRT-PCR and Western blot were applied to investigate the protein and mRNA expression of keratin 5 (CK5), keratin 19 (CK19), kinase insert domain receptor (KDR), and von Willebrand factor (VWF) (Additional file 1: Table S1) in the co-culture system of MS and ADSC.

### Secretion function of microskin

To investigate the secretion ability of microskin, microskin about 1-cm^2^ epidermis area was put in a 6-well plate uniformly and incubated for 3 days, 7 days, and 14 days. The supernatant was taken for EGF and VEGF secretion detection (Elabscience, China) by enzyme-linked immunosorbent assay (ELISA). The concentration of cytokines demonstrated the secretion function of microskin.

### Paracrine analyze by protein array

The cultured microskin suspends and the co-cultured microskin with ADSC suspend were taken as protein array samples. A total of 60 proteins were selected, including growth factors, chemotactic factors, and inflammation factors. All the samples were analyzed using an array (RayBiotech, Norcross, GA, USA, GSH-ANG-1000). All experiments were conducted according to the manufacturer’s instructions. Briefly, after 60 min of incubation with blocking buffer, 60 μl of 100-fold concentrated samples was added to each well. After overnight incubation at 4 °C and extensive washing, the biotin-labeled detection antibody was added for 2 h and then washed away. AlexaFluor 555-conjugated streptavidin was then added and incubated for 1 h at room temperature. The signals (532-nm excitation, 635-nm emission) were scanned and extracted using an InnoScan 300 scanner (Innopsys, Carbonne, France). Raw data from the array scanner were provided as images (.tif files) and spot intensities (tab-delimited.txt file) through Mapix 7.3.1 Software. All experiments were conducted according to the manufacturer’s instructions. Individual array spots were background-subtracted locally and normalized through two positive controls. Calculate the mean signal-BKG for each set of duplicate standards and samples. Then, plot the standard curve on log-log graph paper, with standard concentration on the *x*-axis and signal-BKG on the *y*-axis.

At last, draw the best-fit straight line through the standard points. Concentrations of all serum proteins detected were determined according to its standard curve. It was considered as differentially expressed protein (DEP) by comparison of the signal values between the groups based on *p* < .05 by *t* test, signal value > 150, and fold change (FC) ≥ 1.2 or ≤ 0.83.

### Gene Ontology, KEGG pathway analysis, and integration of protein-protein interaction network analysis

We performed Gene Ontology (GO) analysis and the Kyoto Encyclopedia of Genes and Genomes (KEGG) pathway to analyze the DEPs by using Online String Tools. GO analysis was used to annotate genes and gene products including cellular component, biological process, and molecular function. KEGG was performed for systematic analysis of the pathways in which genes in DEPs were involved in our study by R Bioconductor package clusterProfiler [19].

STRING version 11.0 is a database of protein-protein interactions (PPIs) which covers 5090 organisms. The STRING database is performed to access the protein-protein interactions including direct (physical) and indirect (functional) associations [20]. To evaluate the interrelation among DPEs detected in this study, STRING was utilized and obtained a PPI network through the function and pathway enrichment analysis. It was considered statistically significant with *p* < .05.

### Statistical analysis

Results from the quantitative studies of wound healing analysis in vivo, Western blot, and qRT-PCR were expressed as the mean ± standard deviation (SD). Results from the quantitative studies of the blood vessels in IHC staining were expressed as the mean ± standard error of the mean (SEM). Three independent experiments were performed for validity, and at least three samples per each test were taken for statistical analysis. Statistical comparisons between the two groups were performed by two-tailed Student’s *t* test. Differences among multiple groups were statistically analyzed using one-way analysis of variance (ANOVA). Differences were considered significant when *p* < 0.05.

## Results

### Characterization of ADSCs

According to the Mesenchymal and Tissue Stem Cell Committee of the International Society for Cellular Therapy, we investigated the expression levels of cell surface markers [10]. Flow cytometry analysis indicated that more than 98% of cultured cells expressed CD73 (99.5%), CD90 (98.7%), and CD105 (98.8%), whereas a small fraction of them expressed HLA-DR (1.7%), CD45 (1.8%), CD34 (1.9%), CD19 (0.1%), and CD11b (0.2%) (Additional file 2: Figure S1A). This expression of cell surface markers is a characteristic protein expression of ADSCs.

Differentiation abilities of the isolated cell were further examined by adipogenic, osteogenesis, and chondrogenic. The isolated cells were cultured in special supplemented medium of adipogenic, osteogenesis, and chondrogenic. Staining of Oil Red O (Additional file 2: Figure S1B), Alizarin Red (Additional file 2: Figure S1C), and Alcian blue (Additional file 2: Figure S1D) was carried out to verify the differentiation capacity. The mesenchymal phenotype was supported by their multipotency. All these results demonstrated that the isolated cells were ADSCs.

### Autograft microskin combined with adipose-derived stem cell enhanced wound healing in full-thickness skin defect mouse model

**Fig. 1:**
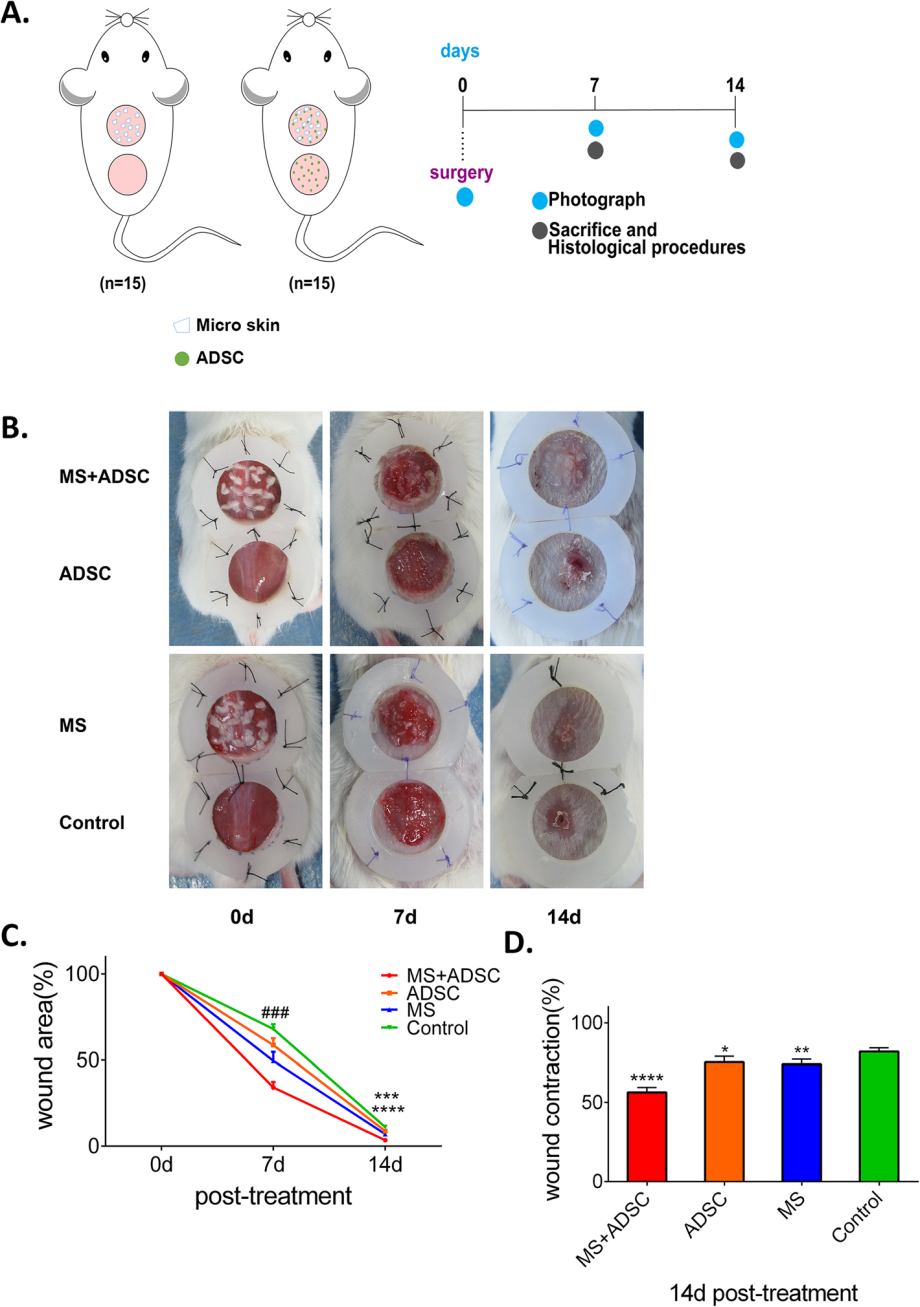
Treatment of ADSC+MS promoted wound healing in a full-thickness skin defect mouse model. **a** Mice were randomized into two parts (*n* = 15 each part). One group accepted the treatment of microskin or PBS, the other accepted microskin plus ADSCs or ADSCs after surgery. Photograph of the wound area was performed at days 0, 7, and 14, and mice were sacrificed to have IHC procedure at days 7 and 14. **b** Representative of a full-thickness wound of each group at day 0, and wound closure can be observed at days 7 and 14 which the MS+ADSC group presented the most remarkable effect of wound healing. **c** Quantitative evaluation of the wound area on days 0, 7, and 14 post-treatment. ^###^*p* < .001, the MS+ADSC group compared to all other groups; ****p < .0001, the MS+ADSC group compared to the ADSC group and control group; ***p < .001, the MS+ADSC group compared to the MS group. **d** Quantitative evaluation of wound contraction at day 14 post-treatment. ****p < .0001; **p < .01; *p < .05, compared to the control group

We originally designed to figure out the effect of microskin combined with ADSCs on cutaneous wound healing in a full-thickness skin defect mouse model. Full-thickness skin wounds were made on the mouse’s back and photographed the open wound area immediately. After surgery, different treatments were performed in the wound area. Besides, the wound area was assessed by photo observation along the time of transplantation and analyzed by photo analyzed software (Fig. 1a). There are no apparent signs of infection of any groups throughout the experiment, including no exudate or purulent drainage. On day 7 post-treatment, the wound area of the MS+ADSC group (34.16% ± 3.113%) was smaller than all other groups (ADSC group, 58.67% ± 3.900%; MS group, 49.55% ± 5.170%; and control groups, 68.05% ± 2.687%; all *p* < .0001). At 14 days after treatment, the wound area of the MS+ADSC group (3.514% ± 1.261%) was almost closed while the other groups (ADSC group, 8.792% ± 0.743%; MS group, 7.039% ± 1.177%; all *p* < .001) still not completely closed with an obviously non-healed area, especially in the control group (11.09% ± 1.324%, *p* < .0001). (Fig. 1b, c). It should be noted that the MS+ADSC group (56.27% ± 3.033%) could suppress the wound contraction on day 14 post-treatment, compared to other groups (ADSC group, 75.29% ± 3.679%; MS group, 73.93% ± 3.224%; and control groups, 81.92% ± 2.380%; all *p* < .0001) (Fig. 1d). Besides, a majority of microskin graft was survival in the newly formed skin coverage close to normal skin in the MS+ADSC group. The newly formed skin was flat without infection and open wound. Besides, there were no signs of hypertrophic scarring, eschar, and hyperpigmentation in the MS+ADSC group. On the contrary, we could observe an obvious wound contraction and eschar in the ADSC group (Additional file 3: Figure S2B), MS group (Additional file 3: Figure S2C), and control group (Additional file 3: Figure S2D). It demonstrated that the treatment of microskin and ADSCs could enhance full-thickness skin defect wound healing in mouse model and suppress wound contraction.

**Fig. 2:**
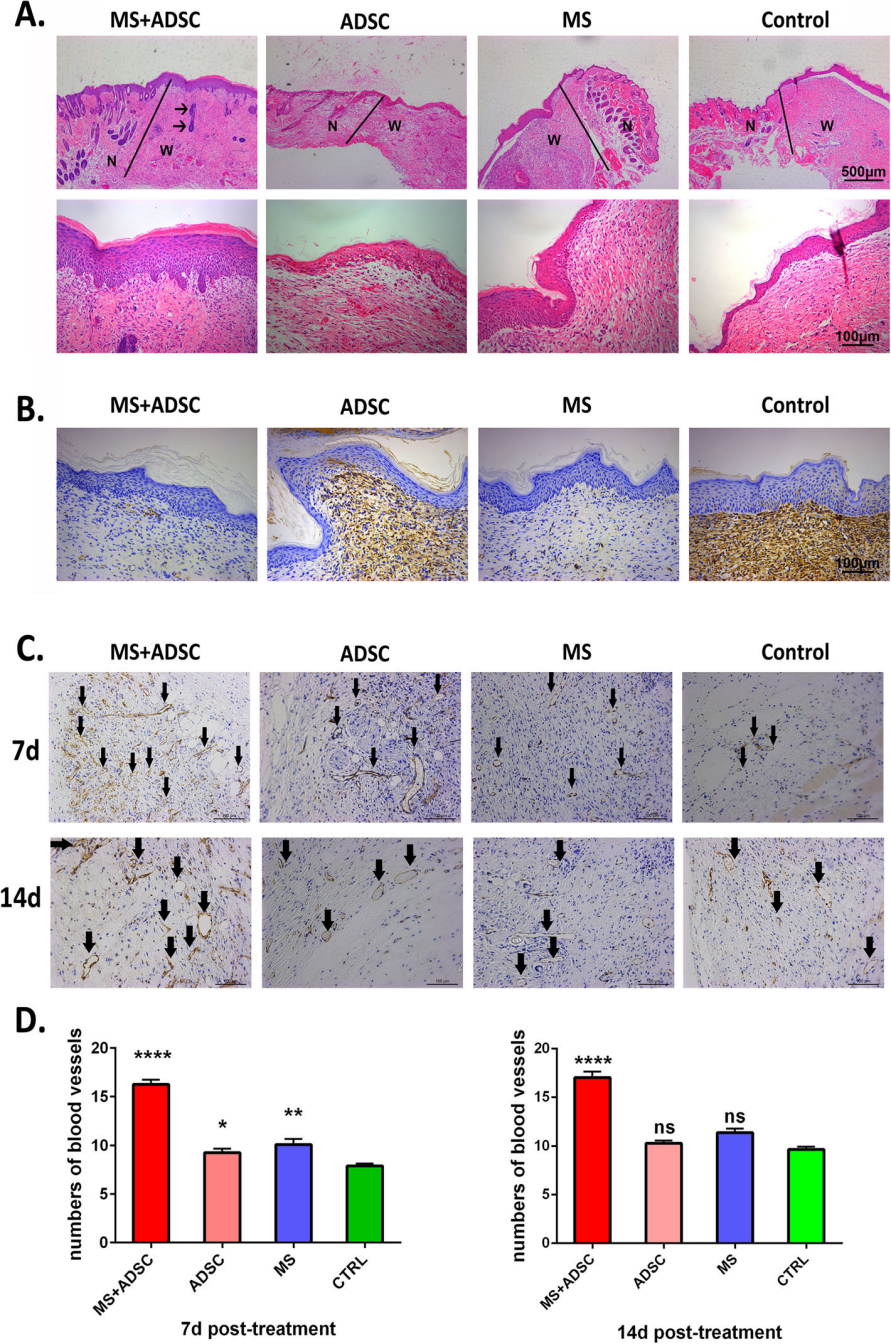
Microscopic appearance of wound beds post-surgery. **a** Hematoxylin and eosin staining of wound beds at day 14 post-surgery. Wounds treated with MS+ADSC showed a newly formed, hyperplastic epithelium that covered the wound area. The black arrows indicate the microskin grafts had become appendages of the newly formed skin. The asterisks indicated normal adnexal structures. “N” is represented for normal skin, and “w” is represented for wound area. **b** α-SMA staining of wound beds at day 14 post-treatment. At day 14, the expression of α-SMA in wound tissue was decreased in the MS+ADSC group compared to others. Scale bars are 100 and 500 μm. **c** CD31 staining of the wound area at days 7 and 14 after treatment. Black arrows indicate CD31-positive vessels. **d** the number of CD31-positive (+) blood vessels per high-power fields (HPFs) (× 20) were quantified to a particular time point. The data expressed are the average means ± SEM, *n* = 5. ****p < .0001, compared to the ADSC group, MS group, and control group; **p < .01; *p < .05; ns, not significant, compared to the control group

In addition, we found a difference between the MS+ADSC group and MS group in hematoxylin and eosin staining. In wound beds at day 14 post-surgery, a newly formed epithelium could be found in the MS+ADSC group with stratum corneum. Besides, the newly formed epithelium of the MS+ADSC group is thicker than the other groups including the MS group. Especially, in the MS+ADSC group, the microskin probably had become appendages of the freshly formed skin, and we seldom observed this phenomenon in the MS group (Fig. 2a). In day 14 post-surgery, also, the MS+ADSC group (0.8143 ± 0.10 mm) presented with a significantly smaller scar thickness than the other groups (ADSC group, 1.00 ± 0.16 mm, *p* < .05; MS group, 1.05 ± 0.13 mm, *p* < .01; control group, 1.24 ± 0.15 mm, *p* < .0001; Additional file 4: Figure S3).

### Combination of ADSCs and microskin suppressed the expression of α-SMA and increased the expression of CD31

IHC staining suggested that the expression of α-SMA in the MS+ADSC group was significantly reduced compared to the MS group and control group, which means the fibrosis in hypodermis was inhibited (Fig. 2b). Neovascularization at the area of the wound was detected by CD31 staining, which was the marker protein of endothelial cells. The representative images of CD31 staining were presented (× 20), and black arrows indicated CD31-positive vessels (Fig. 2c). At day 7, as expectedly, it was shown that the number of new blood vessels in the wound area treated with a combination of microskin and ADSCs (16.27% ± 0.493%) was much higher than the other groups (ADSC group, 9.267% ± 0.431%; MS group, 10.11% ± 0.588%; and control group 7.889% ± 0.247%; Fig. 2d), while there was no significance between the ADSC group and MS group. Although the number of new blood vessels in the ADSC group and MS group was much more than the control group, however, the neovascularization was uneven with a lower density, compared to the MS+ADSC group. At day 14 after treatment, we could observe a large number of mature blood vessel formation in the MS+ADSC group (17.03% ± 0.606%) compared to the other groups (ADSC group, 10.30% ± 0.276%; MS group, 11.40% ± 0.423%; and control group, 9.667% ± 0.27%; Fig. 2d). Notably, there was no significance in both the ADSC group and MS group compared to the control group. These finding indicated that vascular regeneration was better in the MS+ADSC group during full-thickness skin defect regeneration. Therefore, the combination of microskin and ADSCs might accelerate skin wound healing through suppressing fibrosis and promoting vascularization.

### ADSCs did not differentiate into keratinocyte or endotheliocyte cells while co-culturing with microskin

**Fig. 3:**
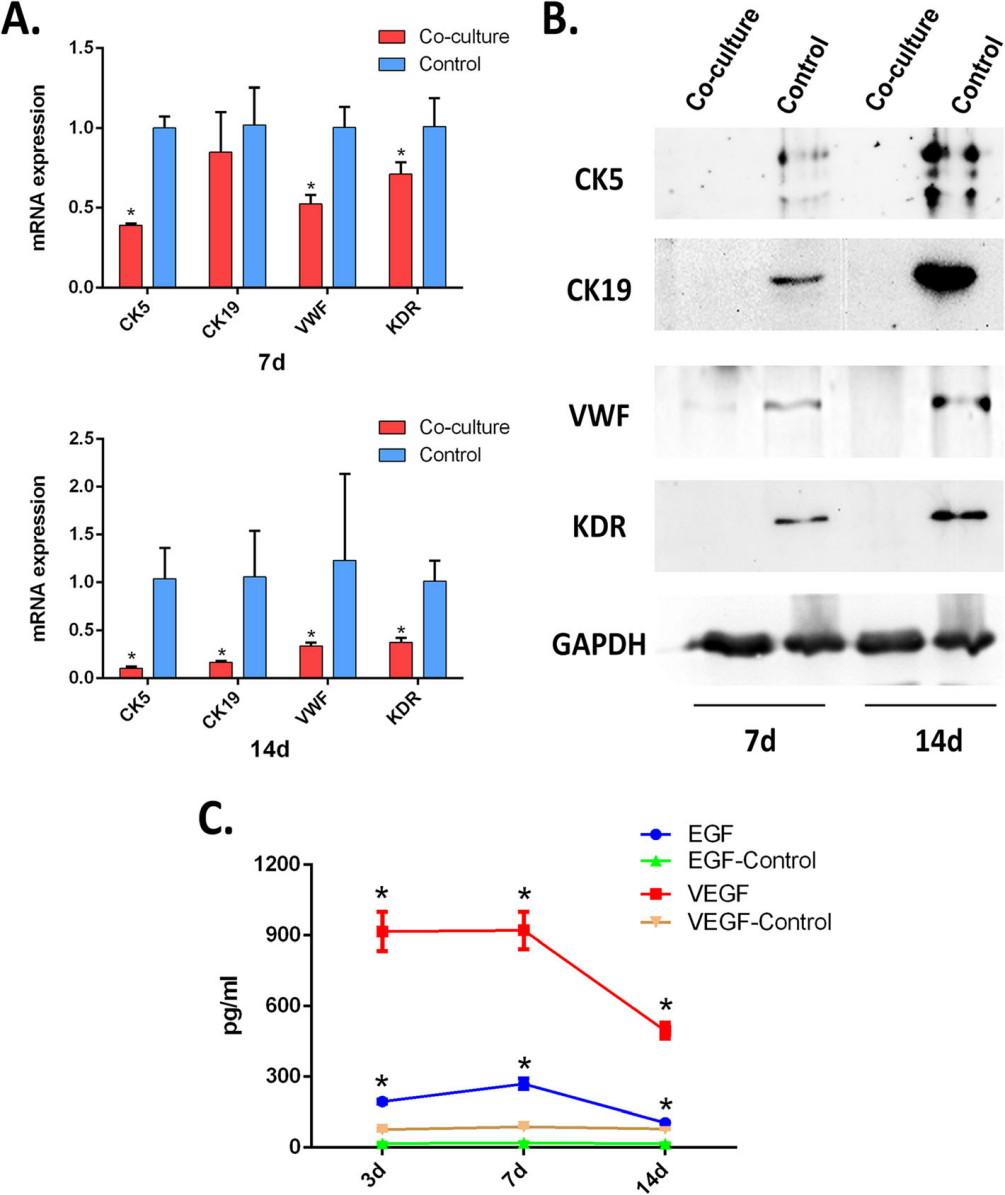
MS+ADSC may enhance wound healing through paracrine function rather than the differentiation of ADSCs. **a** mRNA expression of CK5, CK19, KDR, and VWF are shown in each group. **b** Representative Western blot bands for CK5, CK19, VWF, and KDR expression are shown in each group including at days 7 and 14 post-treatment. In co-cultured system, ADSCs were all downregulated compared to the control group. **c** The concentration of EGF and VEGF secreted by microskin kept stabilizing at 7 days and decreased at 14 days. Both at 7 and 14 days, EGF and VEGF secreted by microskin were higher than the control group (FBS) which demonstrated the microskin could release biological factor. *p < .05, compared with the control group

To investigate whether ADSCs differentiated into keratinocyte or endotheliocyte cells while co-cultured with microskin, we designed a co-culture system as previously described. Western blot and qRT-PCR investigated the expression of CK5, CK19, KDR, and VWF of ADSC in the co-culture system and control ADSC. The protein and mRNA expression of CK5 and CK19 were both downregulated in 7 days and 14 days, along with the downregulation of the protein and mRNA expression of KDR and VWF (Fig. 3a, b). This data demonstrated that the function of ADSC in the co-culture system was not differentiation.

To further identify the secretion ability of microskin, we performed a tissue culture experiment [21]. In our experiment, EGF and VEGF were selected as a reference of secreted cytokines by microskin. At 3 days, 7 days, and 14 days, the secretion of EGF and VEGF was higher than the control group (culture medium) (Fig. 3c). The secretion of EGF and VEGF began declining since the seventh day of culture. These data indicated microskin was able to secrete cytokines in vitro*.*

### Protein array showed several pro-angiogenic cytokines secreted by a co-culture system of ADSC and microskin

**Fig. 4:**
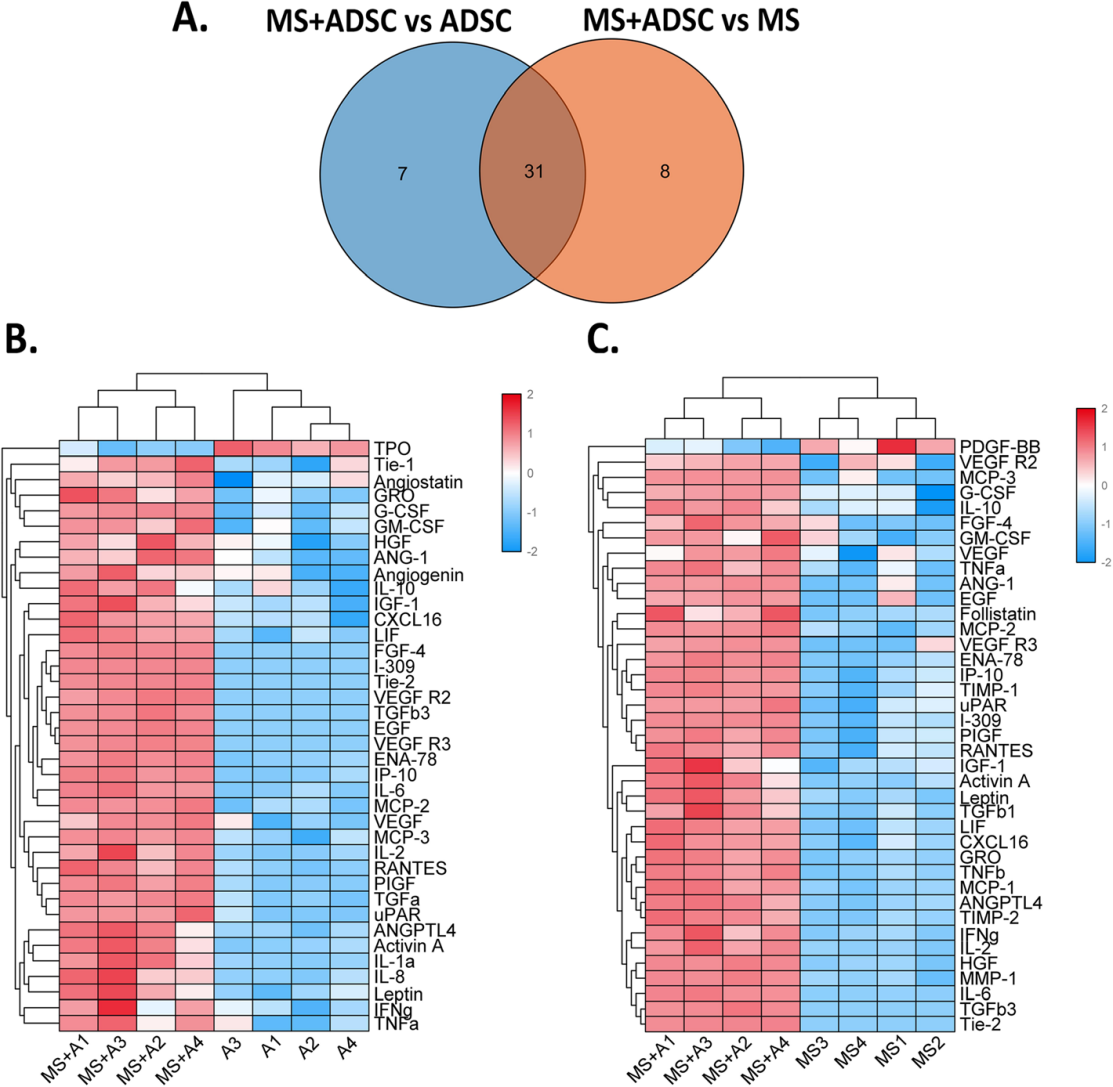
The differentially expressed proteins (DEPs) among the MS+ADSC group, the ADSC group, and the MS group. **a** Venn diagram showed there were 31 common DEPs detected in the MS+ADSC group compared to the ADSC group and MS group. R Bioconductor package and golots were performed to obtain heat map of DEPs in the MS+ADSC group compared with the ADSC group (**b**) and MS group (**c**), with the cluster analysis results

A total of 60 angiogenic cytokines were detected in the culture supernatants of microskin (MS+A group), ADSCs (ADSC group), and microskin (MS group) on day 7. As expected, all three groups highly expressed several growth factors, including angiopoietin-1 (ANG-1), placental growth factor (PIGF), Tie-2, hepatocyte growth factor (HGF), EGF, and VEGF. Specifically, there were 31 common differential cytokines detected in the MS+A group and were upregulated compared to the MS group ADSC group (Fig. 4a). Also, the heat map of differentially expressed proteins and the detailed cluster analysis were presented (Fig. 4b, c). From the heat map, we could find that co-culture of microskin and ADSCs could upregulate the expression of most cytokines detected in vitro compared to culturing microskin and ADSCs only. Generally, there were 29 and 32 upregulated DEPs detected in the MS+ADSC group compared to the ADSC group and MS group, respectively. Also, the top upregulation cytokines detected in the MS+ADSC group were I-309, EGF, G-CSF, Tie-2, and VEGF-R2 in comparison with the ADSC group; while it was Tie-2, MCP-3, HGF, ANG-1, and GSF in comparison with the ADSC group. We could confirm that there was a regulation of biological factors while co-culture of microskin and ADSCs in vitro.

### GO/KEGG enrichment analysis of differentially expressed proteins

**Fig. 5:**
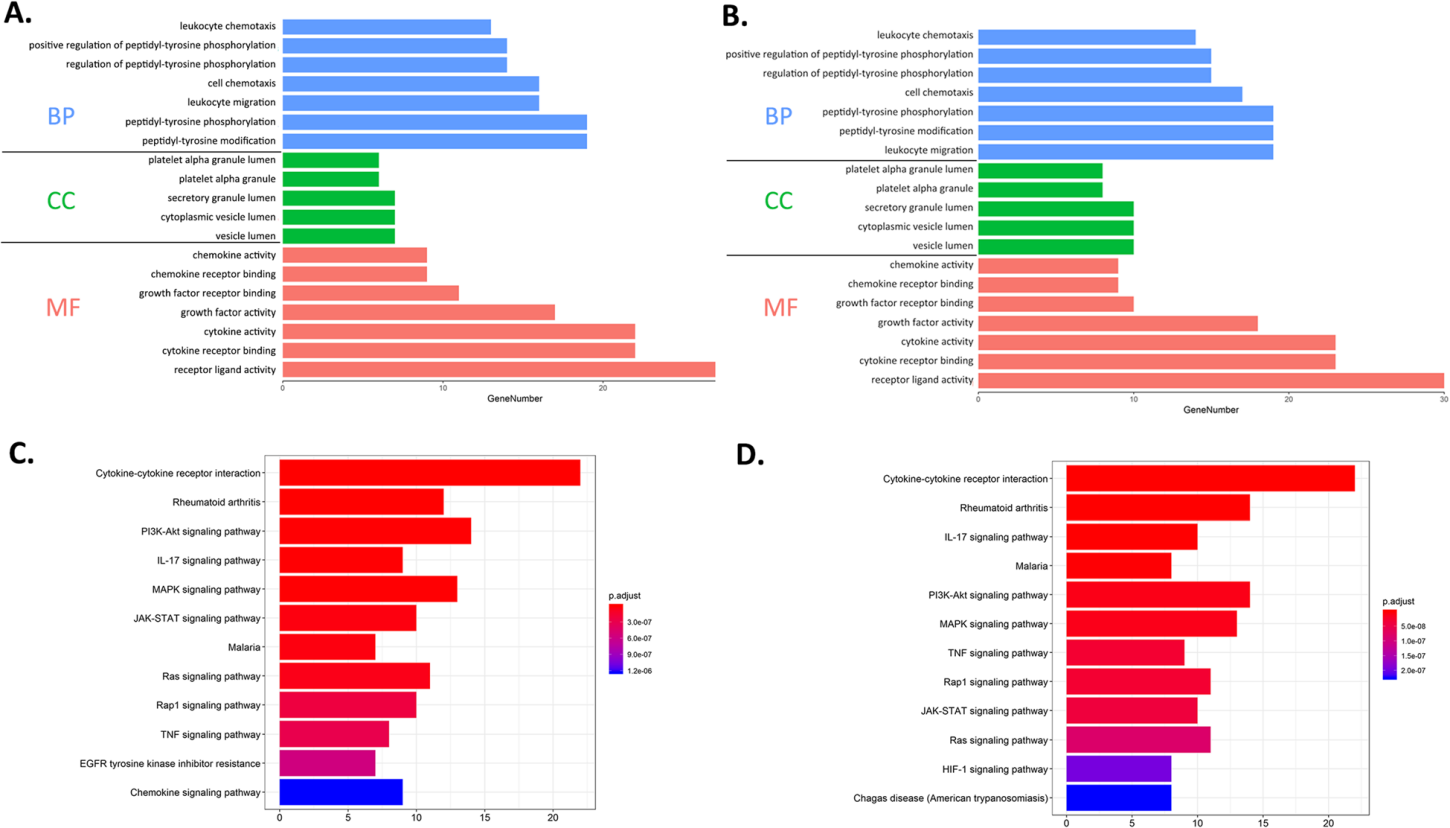
GO/KEGG enrichment analysis of DEPs in the ADSC+MS group compared to the ADSC group and MS group by DAVID online tool and R package clusterProfiler. **a** GO enrichment results of the ADSC+MS group versus ADSC group. **b** GO enrichment results of the ADSC+MS group versus MS group. **c** KEGG pathway enrichment results of the ADSC+MS group versus ADSC group. **d** KEGG pathway enrichment results of the ADSC+MS group versus ADSC group. GO, Gene Ontology; KEGG, Kyoto Encyclopedia of Genes and Genomes

For GO enrichment analysis, the comparison of the MS+A group versus the MS group and MS+A group versus a group showed similar results (Fig. 5a, b). There were three notable cellular component (CC) enrichment terms, including vesicle lumen, cytoplasmic vesicle lumen, and secretory granule lumen. The biological process (BP) analysis indicated that co-culture of microskin and ADSCs mainly had two effects. One was positive regulation of cell migration and cellular component movement, and the other was peptidyl-tyrosine phosphorylation. The molecular function (MF) results showed that the differentially expressed proteins in the MS+A group played critical roles in enhancing the activity of cytokines, growth factors, receptor regulator, and receptor ligand. Also, it made a difference in molecular binding, including cytokine receptor binding, growth factor receptor binding, and G protein-coupled receptor binding.

The results of KEGG analysis of the MS+ADSC group versus the ADSC group and the MS+ADSC group versus the MS group are highly similar (Fig. 5c, d). According to the differentially expressed proteins, KEGG analysis results indicated that PI3K-Akt [22] and Jak-STAT [23] signaling pathways were involved with co-culture of microskin and ADSCs in vitro, which may induce migration and proliferation of fibroblasts and keratinocytes. Besides, PI3K-Akt [24] and Ras [25] signaling pathways were also highly involved with co-culture of microskin and ADSCs in vitro. As a result, the activity of these signaling pathways may promote angiogenesis. The KEGG analysis demonstrated that co-culture of microskin and ADSCs could activate several signaling pathways which were highly related to enhance skin wound healing through angiogenesis and cell migration and proliferation.

### PPI network analysis of the significant associated DEPs

**Fig. 6:**
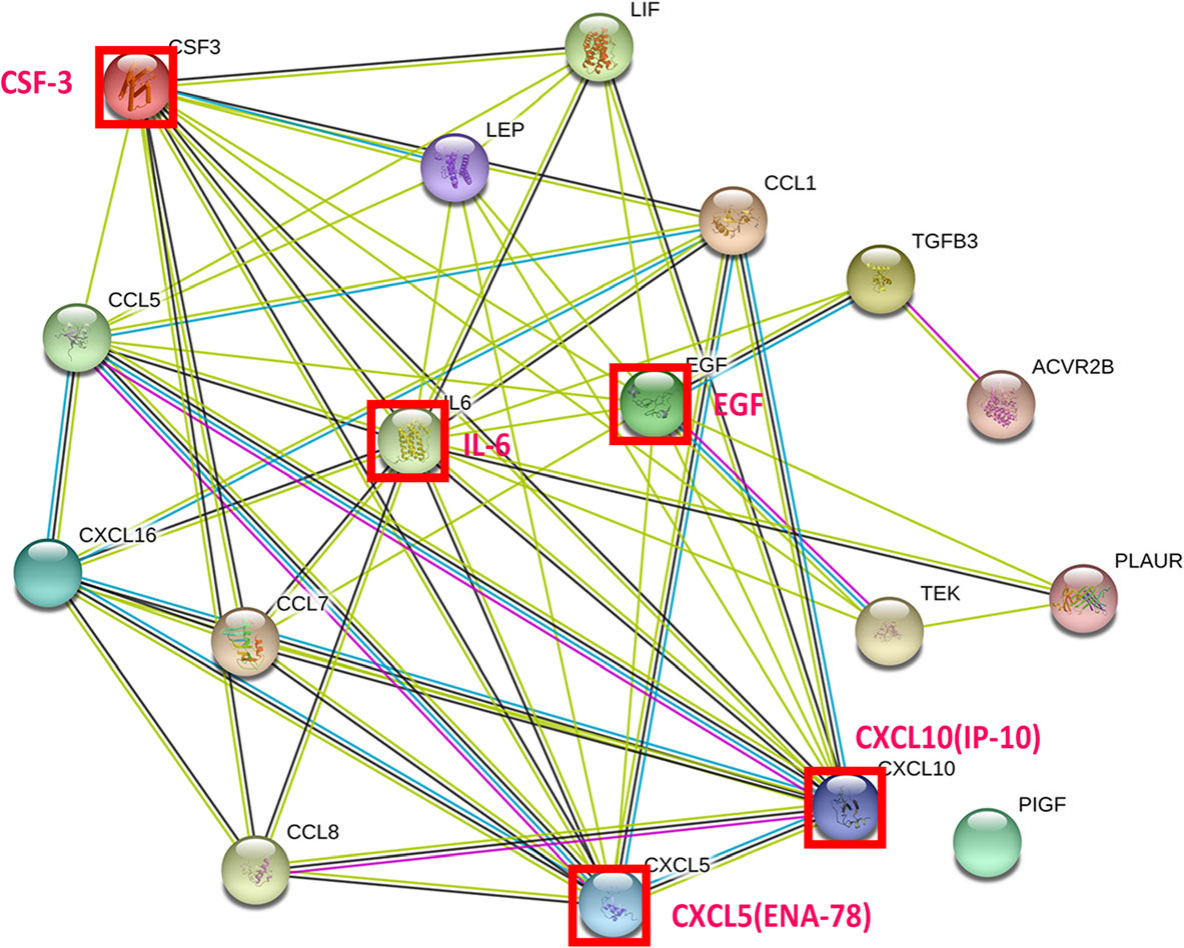
Intermolecular interactions of vital associated differentially expressed proteins (DEPs). IL-6, G-CSF, EGF, IP-10, and ENA-78 held a large number of interactions. The interaction relationship of DEPs was shown as the lines. Network edges: line color represents the type of interaction evidence from the interaction sources, line shape represents the predicted mode of action, and line thickness represents the strength of data support. Colored nodes: query proteins and first shell of interactors; empty nodes: proteins of unknown 3D structure; filled nodes: proteins of some known or predicted 3D structure

To better analyze the protein-protein interaction in our study, a section of common significant associated differentially expressed proteins (FC > 1.5, *p* < .05) was chosen (Table 1). The PPI network included 17 nodes and 57 edges (Fig. 6). In the PPI network, IL-6 (14), G-CSF (13), EGF (11), IP-10 (11), and ENA-78 (10) possessed a large number of interactions (as displayed in the parentheses). Moreover, these proteins might be the core protein in this PPI network of a total of 17 nodes. Therefore, they were considered to be the potential proteins in further studies of the treatment of microskin and ADSCs to full-thickness skin defect.

**Table 1:** The significant associated differentially expressed proteins in the MS+ADSC group (FC > 1.5, *p* < .05)

Entrez ID	Protein	FC (MS+A versus MS)	FC (MS+A versus ADSC)
3624	Activin A	4.34318	4.41328
58,191	CXCL16	2.24848	1.78707
1950	EGF	3.23495	11.55117
6374	ENA-78	6.73969	7.97758
1440	G-CSF	6.57958	11.01831
6346	I-309	7.55954	4.49978
3569	IL-6	8.25032	4.49978
3627	IP-10	6.27871	5.81403
3952	Leptin	2.56586	1.64033
3976	LIF	2.80270	2.75564
6355	MCP-2	4.34065	5.28009
6354	MCP-3	8.42093	4.27534
5228	PIGF	3.20651	3.85779
6352	RANTES	3.03501	2.01860
7043	TGFb3	10.52096	10.52096
7010	Tie-2	10.98094	10.98094
5329	uPAR	2.39436	1.82882
3791	VEGF R2	1.81428	10.66111
2324	VEGF R3	4.88643	11.08758

*Entrez*, Entrez Gene (http://www.ncbi.nlm.nih.gov/gene), *FC* fold change, *MS+A* combination of microskin and ADSCs group, *MS* microskin group, *ADSC*, ADSCs group

## Discussion

For the reconstruction of a large area of wounded skin, there are problems to be solved, including lack of microskin, wound contraction, delayed vascularization, and scar formation [26]. ADSCs were easy to obtain and apply to many clinical trials and make a great result, including skin regeneration, with the ability to regenerate wounded tissue, enhancing vascularization, and inhibiting fibrosis [18]. To our best knowledge, this is the first study of microskin and ADSCs used in full-thickness skin defect mouse model. In the present study, the mouse model showed that this combination of therapy could promote full-thickness skin defects regenerating with faster wound closure and more neovascularization. Our results indicated co-transplantation microskin and ADSCs promote skin tissue regeneration by the secretions of multiple cytokines which enhance pro-angiogenic.

Clinically, autograft microskin transplantation was one of the accepted standard therapies to massive area skin damage [2]. Microskin transplantation had been employed to treatment for extensive skin damage for years [27]. However, there are still some limitations, such as a limited abundance of donors, less revascularization, and failure of transplantation [28]. Evidence supported that ADSCs could prolong the survival of skin grafts with angiogenic effect and ameliorate microcirculatory alterations [29, 30]. In our work, the combination of microskin and ADSCs enhanced the repairing of skin defect with a faster wound healing with less wound contraction, compared to transplantation of ADSCs or microskin only. Interestingly, the wound area at 14 days showed no statistical significance between the MS group (7.039% ± 1.177%) and ADSC group (8.792% ± 0.743%). It should be noted that the survival rate of microskin was increased in the MS+ADSC group compared to the MS group (Additional file 5: Figure S4B). This may reflect that the combination of microskin and ADSCs could better accelerate skin wound healing.

To properly evaluate the therapeutic effect of the combination of microskin and ADSCs, we performed an immunohistochemistry staining of the wounded skin of each group. ADSCs, including their derived extracellular microvesicles, were used to skin tissue regeneration [31]. It is confirmed that ADSC could facilitate angiogenesis in tissue repair [32]. In our study, we observed that the MS+ADSC group showed more neovascularization by CD31 staining and less fibrosis by α-SMA staining than other treatments. Interestingly, several skin appendages were found in the MS+ADSC group, while few for the other groups. All these data indicated that a better regeneration of skin tissue happened in the MS+ADSC group. However, at day 14, the number of neovascularization was not significant among the MS group (11.40% ± 0.423%), ADSC group (10.30% ± 0.276%), and control group (9.667% ± 0.27%) in this study. Treatment with ADSCs or microskin could play a particular role at the early stage of angiogenesis during skin wound healing. But the angiogenesis effect of ADSCs or microskin has no remarkable superiority compared to the control group at the later stage of skin wound healing. The reason might be the paracrine effect of microskin, and ADSCs was weakened along with prolonging the time of culture (Fig. 3c). We have noticed that there was still a signal of neo-vascularization at 14 days post-injury. The VEGF staining of wounded skin at day 14 post-injury showed the strong positive staining was detected in the MS+ADSC group and ADSC group (Additional file 6: Figure S5). In our vitro study, the expression of VEGF in microskin culturing was declined after day 7, and this might be the reason for the weaken VEGF staining of the wound sections at day 14 post-injury in the MS group. Normally, the number of vessels will normalize and return to a level close to the normal skin at the late stage of wound healing [33]. In our study, the larger number of neovascularization in the MS+ADSC group may due to the mutual promotion of cytokine secretion, which was still unclear. The combined impact of microskin and ADSCs showed a distinct advantage in angiogenesis which accelerated concrescence of the skin wound.

We first wonder whether ADSCs could differentiate into epidermal cells or endothelial cells to enhance wound healing in coaction with microskin [16, 17]. The co-culture system of microskin and ADSCs showed that the keratin expression in ADSC was downregulated, along with angiogenesis marker VWF and KDR. In other words, microskin might not accelerate the differentiation ability of ADSCs in our study. However, there were a few research reported the secretion function of skin tissue culture [34]. Therefore, we investigated the paracrine effect of microskin. We observed that microskin could secret several cytokines while cultured in the Transwell system, which was a persistent secretion of EGF and VEGF. However, the secretion was declining since day 7 of culture. The result of our in vivo experiment also reflected that treatment of microskin or ADSCs only might not access a satisfying outcome of angiogenesis.

The paracrine function was another critical function of ADSCs in wound healing. Therefore, we further investigated the two-way paracrine effects between microskin and ADSCs, and the data indicated that combination of microskin and ADSCs could secrete various cytokines with an up-expression, such as VEGF, IL-6, HGF, and EGF (Fig. 4b, c). These cytokines have been confirmed that they play an essential role in promoting angiogenesis [35, 36]. Through GO enrichment analysis, we found that the biological process of DEPs in the MS+ADSC group was involved in cell migration, cellular component movement, and peptidyl-tyrosine phosphorylation. The molecular function (MF) results of them were involved in enhancing the activity of cytokines, growth factors, receptor regulator, and receptor ligand. Moreover, three important cellular component (CC) enrichment terms were detected, including vesicle lumen, cytoplasmic vesicle lumen, and secretory granule lumen. Also, the most significant pathways were enriched in KEGG analysis, which are cytokine-cytokine receptor interaction and PI3K-Akt and MAPK signaling pathways. These signaling pathways were essential to wound repair [22]. The paracrine of DEPs were enriched in biological process and signaling pathway related to growth factors and inflammation. The wound healing process after treatment of microskin and ADSCs might involve the various cellular metabolic process by the DEPs. By binding with other molecules (e.g., cytokines, chemokines, and receptors) and activity of growth factors, receptors, and ligands, they affect cell and cellular component movement, inflammatory response, and phosphorylation. This combination of growth factors and inflammatory factors stimulated wound healing-related cell migration and angiogenesis for tissue regeneration.

Wound healing is a highly complex process and involves a variety of complicated interactions among different resident cells extracellular, extracellular matrix, soluble cytokines, and infiltrating leukocyte subtypes. It has been confirmed that an appropriate inflammatory response is necessary for healthy wound healing [37]. However, the excessive inflammatory response may lead to delay healing or non-healing wounds [38]. In this study, inflammatory factors (e.g., IL-6) and growth factors (e.g., EGF) were upregulated in the heat map and hold a larger number of interacting proteins in the PPI network. IL-6 is considered as an inflammatory factor secreted from cells that induce angiogenesis process in murine skin isografts [39]. With the close correlation of VEGF, treatment of epithelial cell lines with IL-6 can induce the expression of VEGF mRNA to promote angiogenesis [40]. It is reported that IL-6 secreted from ADSCs can stimulate angiogenesis, accelerate cutaneous wound healing, and enhance recovery after ischemia/reperfusion (I/R) injury to increase flap survival [41–43]. In the present study, we found increasing neovascularization in the MS+ADSC group in mouse model and an upregulation of IL-6 in protein array, which was consistent with these previous studies. Our protein array detected a series of upregulation of angiogenic factors, such as IL-6, Tie-2, uPAR, and G-CSF. Besides, IL-6, G-CSF, EGF, IP-10, and ENA-78 might be the core proteins in our study with a large number of interacting proteins. It was probably that the combination of inflammatory factors and growth factors might play a considerable part in angiogenesis.

Meanwhile, we have noticed that over a dozen highly relevant cytokines (FC > 1.5, *p* < .05) were both detected in the MS+ADSC group in the protein array, compared to the ADSC group and MS group (Table 1). It was quite remarkable that a couple of angiogenic cytokines were upregulated, including VEGFR2, G-CSF, Tie-2, and MCP-3. These cytokines were confirmed highly related to angiogenesis by regulating the migration, proliferation, and survival of vascular endothelial cells and upregulating angiogenic cytokines [44–47]. We speculated combination treatment of microskin and ADSCs promoted angiogenesis by upregulating angiogenic cytokines.

Although the underlying mechanisms remain indistinct, our result suggests that the cytokines, including VEGF, IL-6, HGF, and EGF, which were upregulated in supernatants of co-culturing of microskin and ADSCs, may play an essential role during the process of wound healing. Furthermore, our data revealed the regulation of the two-way cytokine between microskin and ADSCs. Most of the cytokines we detected were up-expression in co-culturing of microskin and ADSCs. There should be a positive feedback loop that upregulates the cytokines to promote wound healing. However, future study is needed to find out the critical cytokine and underlying signaling pathway in our study, as well as the target cells. Therefore, we propose a possible mode of clinical translation as follows: A combined transplantation of microskin and ADSCs is operated to the wound site. The cytokines derived from microskin and ADSCs could enhance the wound healing with a better vascularization (Additional file 7: Figure S6).

There is still a limitation related to our study. Our investigation focused on tissue-to-cell and cell-to-tissue interactions, while wound healing including tissue-to-cell, cell-to-tissue, and cell-to-cell interactions. Inflammation cells, extracellular matrix, and blood supply also contributed to wound healing, not only adipose stem cells, fibroblasts, and microskin. Further investigation is needed to figure out which cytokine and signaling pathway are critical in vivo study.

## Conclusions

Our present study demonstrated that autograft microskin combined with adipose-derived stem cell could enhance the healing of a large-area wound in a mouse model with better angiogenesis. The treatment with microskin and ADSCs improve angiogenesis and reduce fibrosis with the secretion of multiple cytokines. The interaction network of various upregulated cytokines secreted by microskin and ADSCs, such as IL-6, G-CSF, EGF, IP-10, and ENA-78, may play an essential role in promoting wound healing. The combination of microskin and ADSCs may be a promising therapy to enhance tissue regeneration of full-thickness skin defect.

## Additional files

Additional file 1: **Table S1.** Primers of quantitative reverse transcription–polymerase chain reaction (qRT-PCR). (DOC 29 kb)

Additional file 2: **Figure S1.** Characterization and differentiation capacity of ADSCs. **(A):** Flowcytometry analysis indicated that more than 98% of cultured cells expressed CD73 (99.5%), CD90 (98.7%) and CD105 (98.8%), whereas a small fraction of them expressed HLA-DR (1.7%), CD 45 (1.8%), CD 34 (1.9%), CD 19 (0.1%) and CD 11b (0.2%). ISO-Alexa Flour, ISO-PE, and ISO-APC were considered as controls (PE: phycoerythrin; APC: allophycocyanin) **(B):** Oil Red O staining of ADSCs cultured in adipogenic media. Scale bar = 50 μm **(C):** Alizarin red staining of ADSCs cultured in osteogenic media. Scale bar = 50 μm **(D):** Alcian blue staining of ADSCs cultured in chondrogenic media. Scale bar = 100μm. (TIF 4594 kb)

Additional file 3: **Figure S2.** Treatment of MS+ADSC obtained cosmetically appealing at day 14 post-injury compared to other groups. **(A):** MS+ADSC group **(B):** ADSC group **(C):**MS group **(D):** control group. (TIF 2180 kb)

Additional file 4: **Figure S3.** Treatment of MS+ADSC reduced scar thickness at day 14 post-injury. **(A):** Representative photomicrographies of the scar tissue which determined on H&E stained sections of wounded skin at day 14 post-injury (dashed lined area), and black lines indicated the scar thickness. **(B):** Scar thickness in tissue sections of day 14 post-injury skin. The data expressed are an average means ± SEM, *n*=5. ****, *p* <.0001; **, *p* <.01; *, *p* <.05, compared to control group. (TIF 6116 kb)

Additional file 5: **Figure S4.** ADSCs play a role in improving the survival rate of micro skin grafts. **(A):** The original area of micro skin grafts (rounded up by yellow line) **(B):** The area of survival micro skin grafts (rounded up by yellow line) **(C):** Representative of survival rate in MS+ADSC group and MS group. The data expressed are an average means ± SEM, *n* = 5. ****, *p* <.0001. (TIF 1249 kb)

Additional file 6: **Figure S5.** VEGF staining of wounded skin at day 7 and 14 post-injury. There was a stronger positive staining of VEGF at wounded skin in MS+ADSC group. Both day 7 and day 14 post-injury, the positive staining was stronger in MS+ADSC group compared to other groups. (TIF 6239 kb)

Additional file 7: **Figure S6.** A possible mode of clinical translation about the combined transplantation of micro skin and ADSCs. After a debridement of massive injury and purchased autologous ADSCs, on the day of surgery, a combined transplantation of micro skin and ADSCs is operated to the wound site. The cytokines derived from micro skin and ADSCs could enhance the wound healing with a better vascularization. (TIF 1798 kb)

## Data Availability

The datasets generated and/or analyzed during the current study are included within the article and are available from the corresponding authors on reasonable request.

## Abbreviations

ADSC: Adipose-derived stem cell
BP: Biological process
CC: Cellular component
DEP: Differentially expressed protein
ECs: Endothelial cell
EGF: Epidermal growth factor
ELIZA: Enzyme-linked immunosorbent assay
EVs: Extracellular vesicles
FC: Fold change
GO: Gene Ontology
G-CSF: Granulocyte colony-stimulating factor
HGF: Hepatocyte growth factor
HMECs: Human microvascular endothelial cells
KDR: Kinase insert domain receptor
KEGG: Kyoto Encyclopedia of Genes and Genomes
MF: Molecular function
IHC: Histochemistry and immunohistochemistry
IL-6: Interleukin 6
MCP-3: Monocyte chemotactic protein 3
PPI: Protein-protein interactions
Tie-2: Tyrosine kinase with immunoglobulin-like and EGF-like domains 2
uPAR: Urokinase plasminogen activator receptor
VEGF: Vascular endothelial growth factor
VWF: von Willebrand factor
α-SMA: Alpha smooth muscle actin
