# Supplementary material for: Autograft microskin combined with adipose-derived stem cell enhances wound healing in a full-thickness skin defect mouse model
Source: Stem Cell Res Ther. 2019 Aug 30;10:279. doi: 10.1186/s13287-019-1389-4 (PMC6717360; doi:10.1186/s13287-019-1389-4)
Supplement: Supplementary file 1 — Table S1. Primers of quantitative reverse transcription–polymerase chain reaction (qRT-PCR). (DOC 29 kb) [file 13287_2019_1389_MOESM1_ESM.doc]

|  | Forward primer(5' to 3') | Reverse primer(5' to 3") |
| --- | --- | --- |
| KDR | TCATCCTTACCAATTCCCATTTC | ACTGGTAGGAATCCACAGGACA |
| VWF | TCTTCCAGGACTGCAACAAG | TCCGAGATGTCCTCCACATA |
| CK19 | GCGGCCAACGGCGAGCTA | GCAGGACAATCCTGGAGTTCTC |
| CK5 | TGAACACCAAGCTGGCCCTGGA | CTTCCACTGCTACCTCCGGC |

Primers of Mus musculus used in qPCR

KDR, kinase insert domain receptor; VWF, von Willebrand factor; CK19, keratin 19; CK5, keratin 5.
