# Supplementary figures and images for: Autograft microskin combined with adipose-derived stem cell enhances wound healing in a full-thickness skin defect mouse model
Source: Stem Cell Res Ther. 2019 Aug 30;10:279. doi: 10.1186/s13287-019-1389-4 (PMC6717360; doi:10.1186/s13287-019-1389-4)

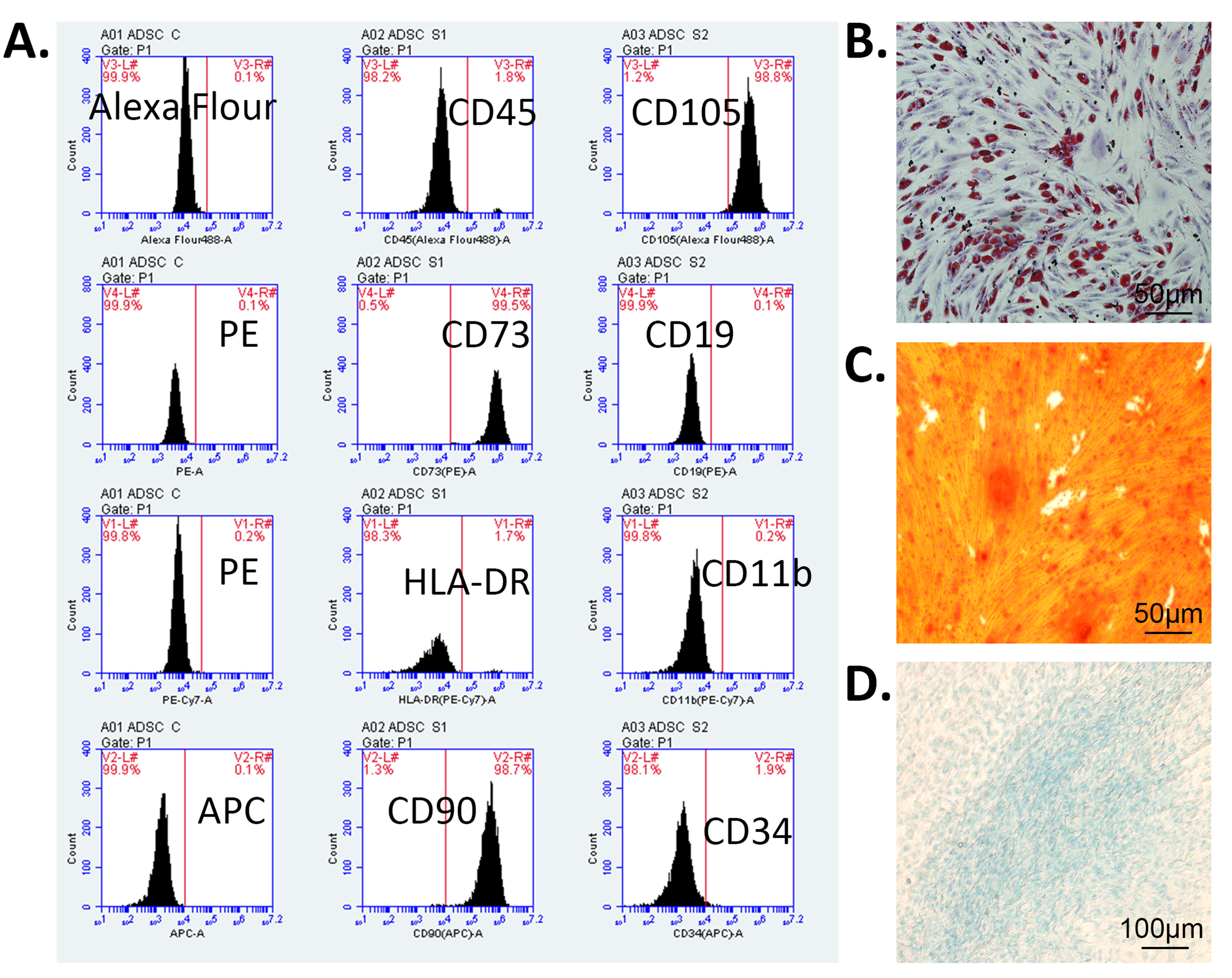

Supplement: Supplementary file 2 — Figure S1. Characterization and differentiation capacity of ADSCs. (A): Flowcytometry analysis indicated that more than 98% of cultured cells expressed CD73 (99.5%), CD90 (98.7%) and CD105 (98.8%), whereas a small fraction of them expressed HLA-DR (1.7%), CD 45 (1.8%), CD 34 (1.9%), CD 19 (0.1%) and CD 11b (0.2%). ISO-Alexa Flour, ISO-PE, and ISO-APC were considered as controls (PE: phycoerythrin; APC: allophycocyanin) (B): Oil Red O staining of ADSCs cultured in adipogenic media. Scale bar = 50 μm (C): Alizarin red staining of ADSCs cultured in osteogenic media. Scale bar = 50 μm (D): Alcian blue staining of ADSCs cultured in chondrogenic media. Scale bar = 100μm. (TIF 4594 kb) [file 13287_2019_1389_MOESM2_ESM.tif]

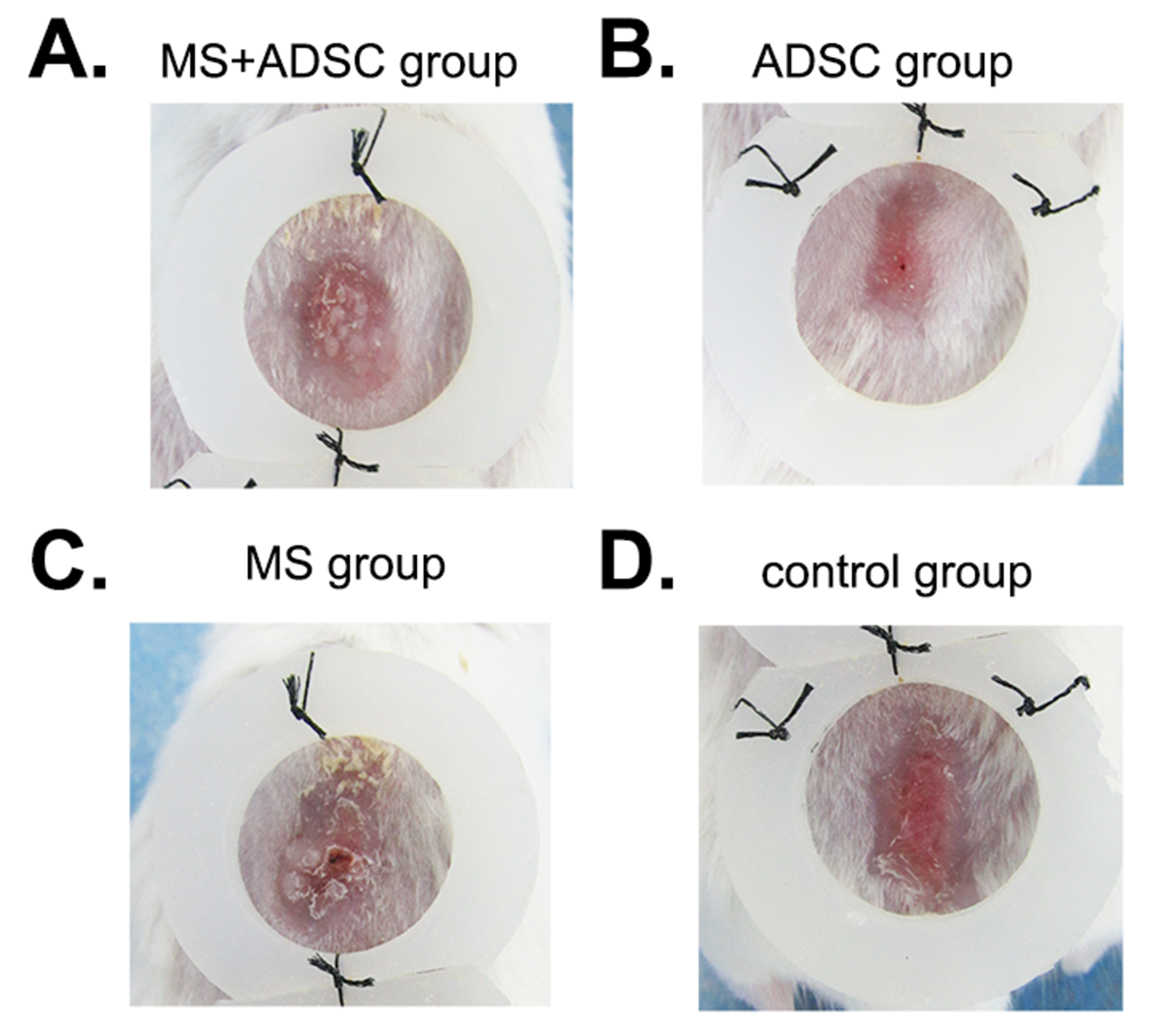

Supplement: Supplementary file 3 — Figure S2. Treatment of MS+ADSC obtained cosmetically appealing at day 14 post-injury compared to other groups. (A): MS+ADSC group (B): ADSC group (C):MS group (D): control group. (TIF 2180 kb) [file 13287_2019_1389_MOESM3_ESM.tif]

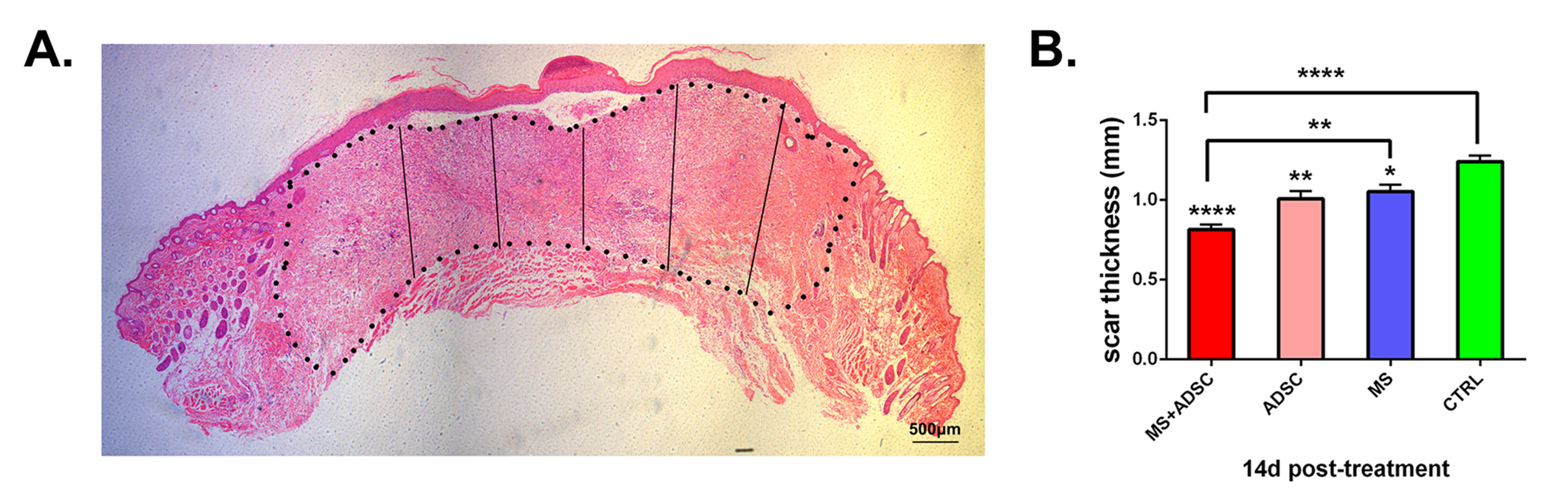

Supplement: Supplementary file 4 — Figure S3. Treatment of MS+ADSC reduced scar thickness at day 14 post-injury. (A): Representative photomicrographies of the scar tissue which determined on H&E stained sections of wounded skin at day 14 post-injury (dashed lined area), and black lines indicated the scar thickness. (B): Scar thickness in tissue sections of day 14 post-injury skin. The data expressed are an average means ± SEM, n=5. ****, p <.0001; **, p <.01; *, p <.05, compared to control group. (TIF 6116 kb) [file 13287_2019_1389_MOESM4_ESM.tif]

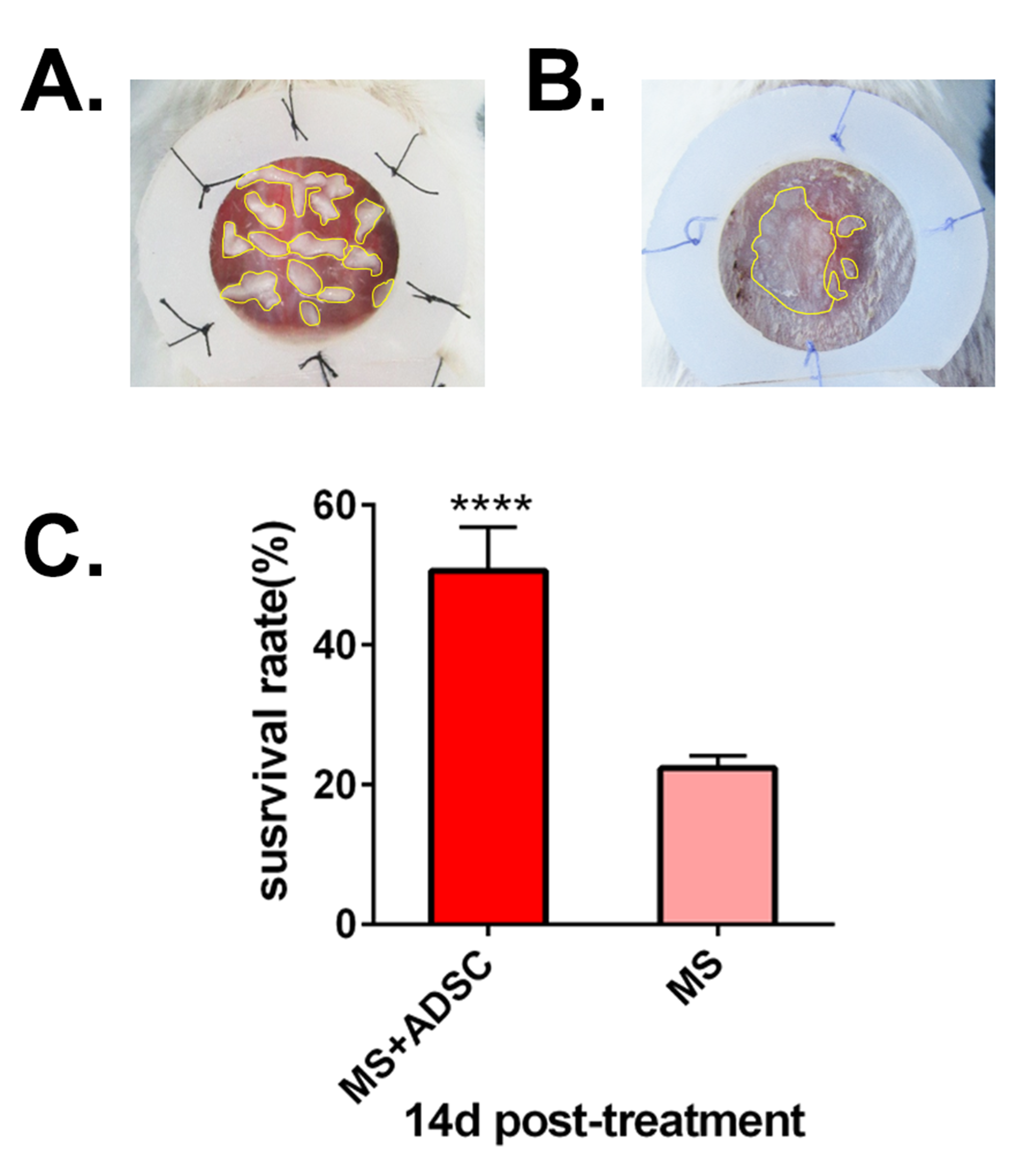

Supplement: Supplementary file 5 — Figure S4. ADSCs play a role in improving the survival rate of micro skin grafts. (A): The original area of micro skin grafts (rounded up by yellow line) (B): The area of survival micro skin grafts (rounded up by yellow line) (C): Representative of survival rate in MS+ADSC group and MS group. The data expressed are an average means ± SEM, n = 5. ****, p <.0001. (TIF 1249 kb) [file 13287_2019_1389_MOESM5_ESM.tif]

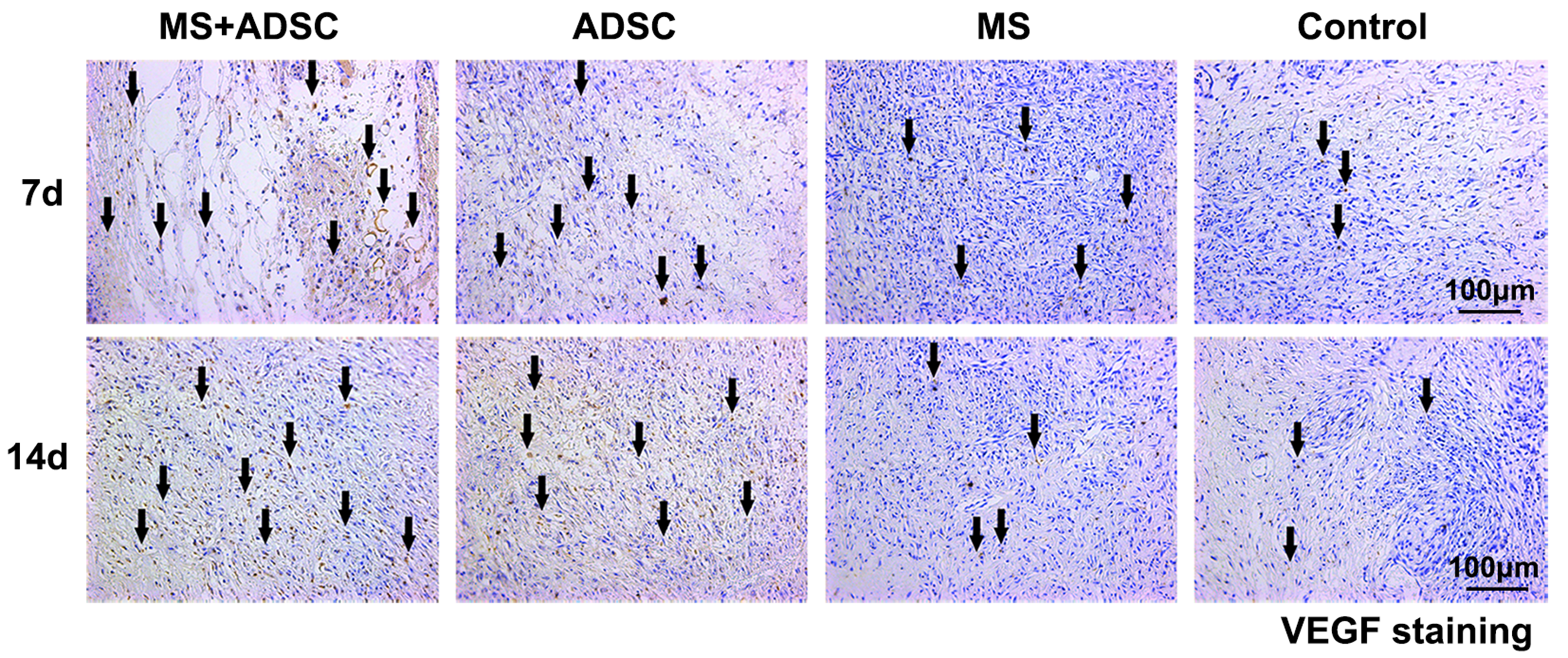

Supplement: Supplementary file 6 — Figure S5. VEGF staining of wounded skin at day 7 and 14 post-injury. There was a stronger positive staining of VEGF at wounded skin in MS+ADSC group. Both day 7 and day 14 post-injury, the positive staining was stronger in MS+ADSC group compared to other groups. (TIF 6239 kb) [file 13287_2019_1389_MOESM6_ESM.tif]

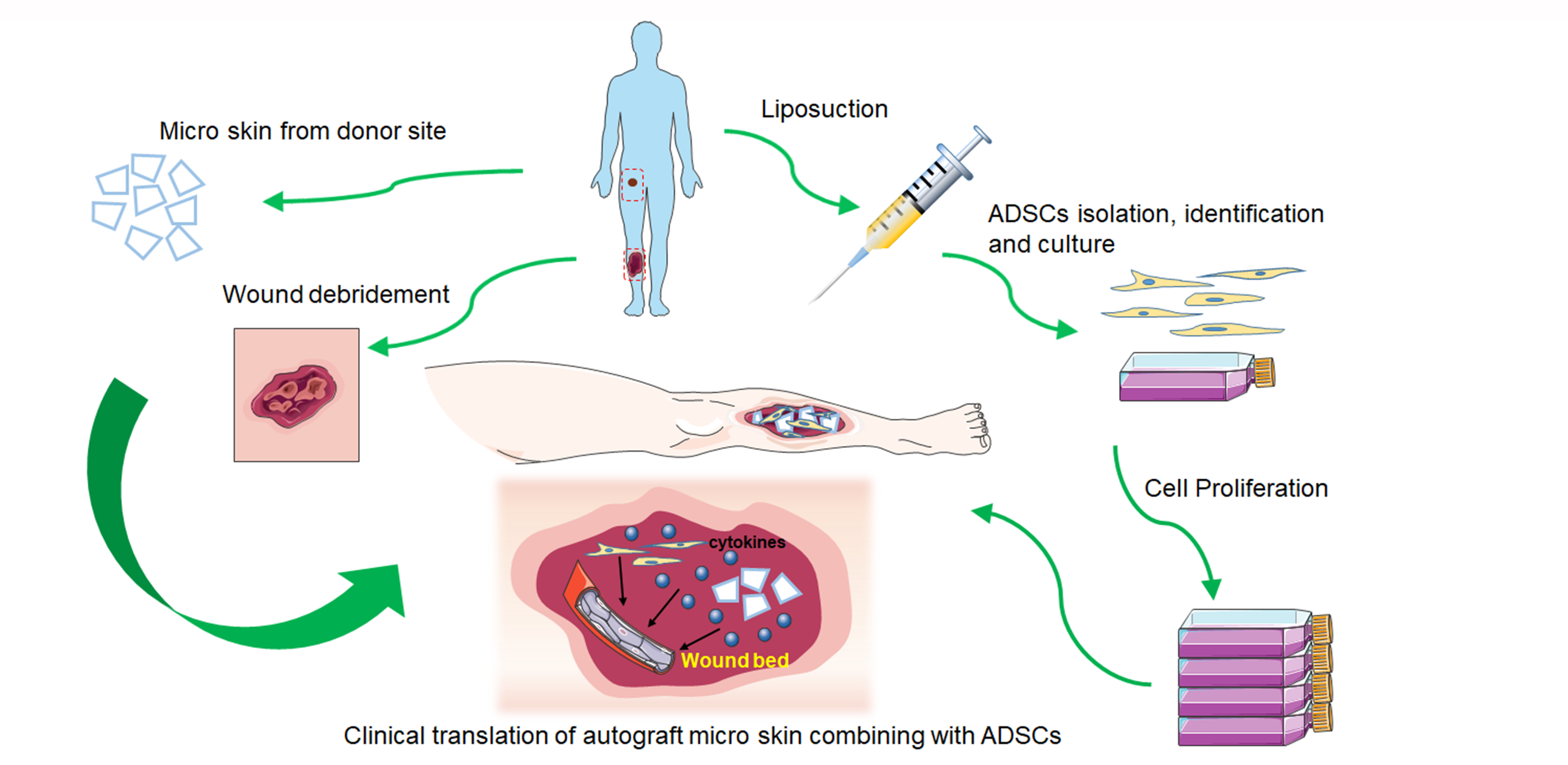

Supplement: Supplementary file 7 — Figure S6. A possible mode of clinical translation about the combined transplantation of micro skin and ADSCs. After a debridement of massive injury and purchased autologous ADSCs, on the day of surgery, a combined transplantation of micro skin and ADSCs is operated to the wound site. The cytokines derived from micro skin and ADSCs could enhance the wound healing with a better vascularization. (TIF 1798 kb) [file 13287_2019_1389_MOESM7_ESM.tif]
